# Non-invasive assessment of arterial stiffness using oscillometric blood pressure measurement

**DOI:** 10.1186/1475-925X-11-6

**Published:** 2012-02-10

**Authors:** Hidehiko Komine, Yoshiyuki Asai, Takashi Yokoi, Mutsuko Yoshizawa

**Affiliations:** 1 Human Technology Research Institute, National Institute of Advanced Industrial Science and Technology (AIST), Higashi 1-1-1, Tsukuba, Ibaraki 305-8566, Japan

**Keywords:** arterial stiffness, blood pressure measurement, pulse wave velocity

## Abstract

**Background:**

Arterial stiffness is a major contributor to cardiovascular diseases. Because current methods of measuring arterial stiffness are technically demanding, the purpose of this study was to develop a simple method of evaluating arterial stiffness using oscillometric blood pressure measurement.

**Methods:**

Blood pressure was conventionally measured in the left upper arm of 173 individuals using an inflatable cuff. Using the time series of occlusive cuff pressure and the amplitudes of pulse oscillations, we calculated local slopes of the curve between the decreasing cuff pressure and corresponding arterial volume. Whole pressure-volume curve was derived from numerical integration of the local slopes. The curve was fitted using an equation and we identified a numerical coefficient of the equation as an index of arterial stiffness (Arterial Pressure-volume Index, API). We also measured brachial-ankle (baPWV) PWV and carotid-femoral (cfPWV) PWV using a vascular testing device and compared the values with API. Furthermore, we assessed carotid arterial compliance using ultrasound images to compare with API.

**Results:**

The slope of the calculated pressure-volume curve was steeper for compliant (low baPWV or cfPWV) than stiff (high baPWV or cfPWV) arteries. API was related to baPWV (*r *= -0.53, *P *< 0.05), cfPWV (*r *= -0.49, *P *< 0.05), and carotid arterial compliance (*r *= 0.32, *P *< 0.05). A stepwise multiple regression analysis demonstrated that baPWV and carotid arterial compliance were the independent determinants of API, and that API was the independent determinant of baPWV and carotid arterial compliance.

**Conclusions:**

These results suggest that our method can simply and simultaneously evaluate arterial stiffness and blood pressure based on oscillometric measurements of blood pressure.

## Introduction

Arterial stiffness is a major contributor to cardiovascular disease [1-3], while we and other groups have demonstrated that regular endurance exercise or diet control improves arterial stiffness [4-7]. Thus the early detection and the daily control of arterial stiffness is a focal point to prevent cardiovascular disease. Arterial stiffness should be assessed in daily living as well as clinic accordingly. Currently, pulse wave velocity (PWV) using applanation tonometry, or arterial compliance using ultrasonography and applanation tonometry are used to assess arterial stiffness. Although these techniques are clinically and experimentally accepted, simpler and easier methodologies are required because the application of tonometry transducers or ultrasound probes on target arteries can be rather difficult.

We therefore aimed to develop a simple and non-invasive method of evaluating arterial stiffness using oscillometric measurements of blood pressure. When blood pressure is measured by cuff oscillometry, brachial arterial volume decreases with inflation of the cuff wrapped around the upper arm, and the arterial volume returns with cuff deflation. Theoretical studies using computer simulation suggest that the relationship between the decreasing cuff pressure (increasing intra-arterial pressure) and the corresponding brachial arterial volume can be described as a sigmoid curve [8-10]. Additionally, the slope of the curve is steeper in a compliant than that in stiff artery [10]. Previous studies using isolated arteries from animals and humans have also demonstrated that elastic property of the arterial wall can be evaluated by relationships between intra-arterial pressure and arterial volume [11-17]. If the curve relationship between cuff pressure and arterial volume during cuff deflation was actually analyzed, arterial stiffness could be assessed as the slope of the curve.

The purpose of this study was to develop a method of evaluating arterial stiffness by assessing the curve between cuff pressure and arterial volume using oscillometric blood pressure measurement. We then validated the method by comparison with PWV and arterial compliance. This novel methodology allows simple and simultaneous measurements of blood pressure and arterial stiffness.

## Methods

### Subjects

We studied 173 healthy volunteers (89 males and 84 females) aged 22 to 75 (48 ± 1) years. The purpose and procedures of this study were explained to the volunteers, who then provided written informed consent to participate. The Ethics Committee of the Institute for Human Science and Biomedical Engineering of the National Institute of Advanced Industrial Science and Technology reviewed and approved the study protocols.

### Curve relationship between cuff pressure and arterial volume

**Figure 1:**
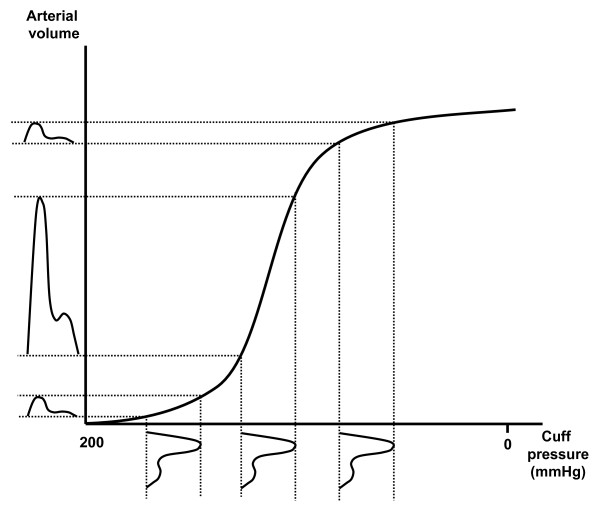
**Relationship between cuff pressure and arterial volume during cuff deflation**. When blood pressure pulses are generated at different cuff pressures as shown below the horizontal axis, corresponding blood volume pulses or cuff oscillations can be described as shown on the left of the vertical axis.

Computer simulations have suggested that the relationship between cuff pressure and arterial volume during cuff deflation can be described as a sigmoid curve (Figure 1) [8-10] and that blood volume pulses or cuff oscillations can be generated by transforming blood pressure pulses [8]. When blood pressure pulses are generated at different cuff pressures, as shown below the horizontal axis of Figure 1, corresponding blood volume pulses or cuff oscillations can be described, as shown on the left of the vertical axis. Based on this theory, local slopes of the pressure-volume curve can be calculated from the occlusive cuff pressure for pulse pressure (systolic - diastolic blood pressure) and the amplitude of cuff oscillations. Thus, we calculated local slopes from cuff pressure and cuff oscillations, and obtained pressure-volume curves by numerically integrating the local slopes.

**Figure 2:**
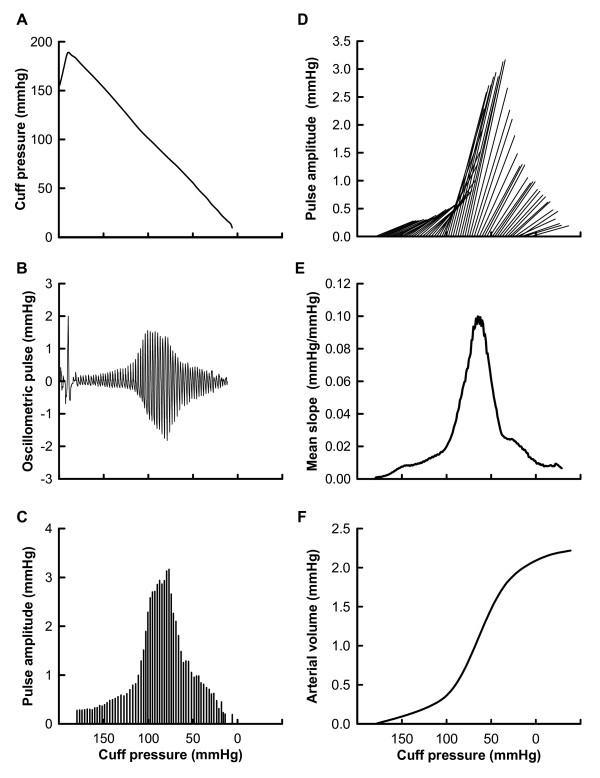
**Procedure to obtain curves between cuff pressure and arterial volume**. A. Time series of occlusive cuff pressure determined after applying a digital filter (low-pass filter at 0.5 Hz) to raw cuff pressure. B. Cuff oscillation evoked by blood pressure pulse obtained after applying a digital filter (band-pass filter of 0.5 - 10 Hz) to raw cuff pressure. C. Amplitudes of cuff oscillations of all blood pressure pulses. D. Local slopes of curves between cuff pressure and arterial volume obtained from amplitudes of all pulse oscillations and changes in cuff pressure for pulse pressure. E. Average of local slopes of curve at all cuff pressure values. F. Pressure-volume curve calculated from numerical integration of averaged slopes.

In the experimental day, a conventional blood pressure cuff was wrapped around the left upper arm of seated participants and was inflated to 190 mmHg at 10 mmHg/s, and deflated to 10 mmHg at 3 mmHg/s. Cuff pressure during inflation and deflation measured using a pressure transducer was stored in a computer at a sampling frequency of 1 kHz for off-line analysis. After recording cuff pressure, a low-pass filter with a cutoff frequency of 0.5 Hz was applied to the raw cuff pressure, and a time series of occlusive cuff pressure was obtained (Figure 2A). A band-pass filter of 0.5 - 10 Hz was applied to the raw cuff pressure to determine cuff oscillation evoked by the blood pressure pulse (Figure 2B). The amplitudes of the cuff oscillations of every blood pressure pulse were calculated (Figure 2C). Heart rate and blood pressure were measured oscillometrically and pulse pressure was calculated from systolic and diastolic blood pressure. Using the amplitudes of all pulse oscillations and changes in the cuff pressure for pulse pressure from the pressure point evoked by the pulses, we calculated the local slopes of the curve between the cuff pressure and arterial volume (Figure 2D). The slopes at all cuff pressures were averaged to estimate the slopes at an arbitrary point on pressure-volume curves (Figure 2E). We calculated the numerical integration of the averaged slopes to generate pressure-volume curves (Figure 2F) that were fitted using the following equation to determine their characteristics:

F(x)=Aarctan(Bx+C)+D

The numerical coefficient B of above equation was used to evaluate arterial stiffness because coefficient B closely reflected the slope of the curve. We identified the numerical coefficient B as an arterial stiffness index and named it the arterial pressure-volume index (API). Cuff pressure was measured three times and the average API was calculated for each participant. The day-to-day coefficient of variation for API in a pilot study on 8 subjects (4 male and 4 female, 22 - 55 years) was 6.0 ± 1.1%.

We measured the circumference of the left upper arm, because we indirectly estimated the arterial pulse volume via the cuff oscillation and thus should examine the effects of the fat or muscle size on API.

### Pulse wave velocity (PWV)

We measured brachial-ankle (baPWV) and carotid-femoral (cfPWV) pulse wave velocity using a vascular testing device (Form PWV/ABI, Omron Healthcare, Kyoto, Japan) as we described [18]. Carotid and femoral arterial pressure waveforms were stored for 30 s using applanation tonometry sensors attached to the left common carotid artery and left common femoral artery. Bilateral brachial and post-tibial arterial pressure waveforms were stored for 10 s by occlusion/sensing cuffs adapted to both arms and ankles. The waveform analyzer measured the time intervals between the carotid and femoral arterial pressure wave (Tcf), and between the brachial and post-tibial arterial pressure wave (Tba). The arterial pressure wave was identified as the start of the sharp systolic upstroke, which was automatically detected using a band-pass filter (5 - 30 Hz). The path length from the carotid to the femoral artery (Dcf) was directly assessed in duplicate with a random zero length measurement over the surface of the body with a nonelastic tape measure [19]. The path lengths from the heart to the brachial artery (Dhb), from the heart to the femur (Dhf), and from the femur to the ankle (Dfa) were automatically calculated in cm using the following equations [20]:

Dhb=(0.220×height-2.07)

Dhf=(0.564×height-18.4)

Dha=(0.249×height-30.7),

where height is in cm,

and then baPWV and cfPWV were calculated as:

baPWV=(dhf+Dfa-Dhb/Tba)

cfPWV=Dcf/Tcf

### Carotid arterial compliance

We also compared the API with carotid arterial compliance, although the sample size was limited to 92 of the 173 participants. Carotid arterial compliance was determined using a combination of ultrasound imaging of the common carotid artery and carotid arterial blood pressure as we described [21]. Carotid arterial blood pressure was noninvasively measured using applanation tonometry (Form PWV/ABI, Omron Healthcare, Kyoto, Japan). Longitudinal B-mode images of the right common carotid artery were obtained ultrasonically (SonoSite 180PLUS, SonoSite Inc., Bothell, WA, USA) with a high-resolution linear-array transducer (10 MHz) placed at 1 to 2 cm proximal to the carotid bulb, with an approximately 90° angle to the vessel so that the near and far wall interfaces were clearly discernible. The ultrasound images were recorded on digital videotapes for offline analysis. The ultrasound images were stored in a computer at 30 Hz and analyzed using image-analysis software (ImageJ 1.32J, NIH, Bethesda, MD, USA). One investigator who was blinded to the subject characteristics performed all image analyses. Carotid arterial lumen diameter was determined as the distance between the vessel far-wall boundary corresponding to the interface between the lumen and intima. The near-wall boundary was defined as the interface of the adventitia and media at minimal diastolic relaxation and at maximal systolic expansion of the vessel. Arterial lumen diameter at minimal diastolic relaxation and maximal systolic expansion of the vessel were measured at three points per video frame and then averaged. Each parameter was averaged over 10 - 15 continuous beats and statistically analyzed. Arterial compliance was determined using the equation:

(CSAs - CSAd)/ΔP,

where CSAs and CSAd are cross-sectional areas at the maximal systolic expansion and at the minimal diastolic relaxation of the carotid artery, and ΔP is the carotid arterial pulse pressure. In addition to arterial compliance, the β-stiffness index was analyzed using the equation:

ln(cSAP/cDAP)×D/ΔD,

where cSAP and cDAP are systolic and diastolic carotid arterial blood pressure, D is end-diastolic carotid lumen diameter and ΔD is the change in carotid lumen diameter between end diastole and peak systole [22]. The β-stiffness index provided an index of arterial compliance adjusted for distending pressure [22].

### Experimental protocol

Subjects abstained from food and caffeine for at least 4 h before the experiment. Measurements were taken in a quiet, temperature-controlled room (24 - 26°C). API, PWV and carotid arterial compliance were measured on the same day in random order.

### Statistical analysis

The relationships between baPWV, cfPWV, carotid arterial compliance, or age and the API were analyzed using a linear regression model and Pearson's correlation coefficient.

To determine which factor explained a given dependent variable, a stepwise multiple regression analysis was carried out. The selected parameters for the analysis were API, baPWV, carotid arterial compliance, mean arterial blood pressure (MAP), and the circumference of the left upper arm. Dependent variables were API, baPWV, or carotid arterial compliance. Independent variables were all of the rest. Parameters were selected with a caution of multicollinearity among the independent variables. Consequently, cfPWV and age were excluded, because cfPWV was strongly correlated with baPWV (*r *= 0.85) and age was significantly related with all of the parameters of arterial stiffness (baPWV, carotid compliance, and API) and MAP.

Statistical significance was defined at *P *< 0.05 for all data, which are expressed in the text and figures as means ± SE.

## Results

The averaged oscillometric arterial blood pressure values were as follows: systolic, 123 ± 1 mmHg; mean, 93 ± 1 mmHg; diastolic, 75 ± 1 mmHg; and the average heart rate was 65 ± 1 beats/min. The averages baPWV and cfPWV were 1333 ± 16 cm/s and 879 ± 12 cm/s, respectively, and the average API was 0.0408 ± 0.0006 U. The coefficient of variation of replicate measurements at the experimental day (intra individual of three time measurements) for API was 7.5 ± 6%.

**Figure 3:**
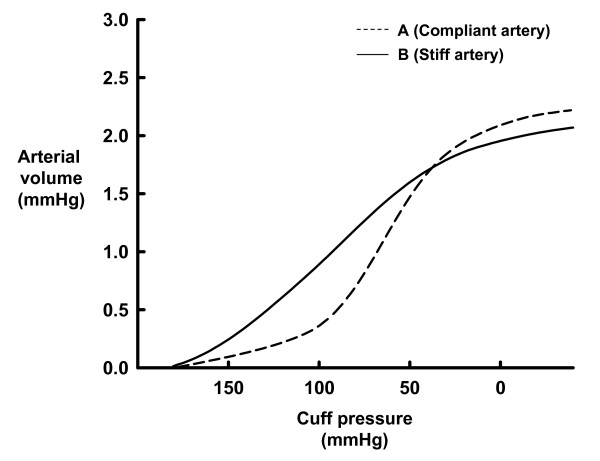
**Relationship between cuff pressure and arterial volume during cuff deflation in two individuals**. A (----) and B (—) represent participants with relatively compliant (baPWV, 1079 cm/s; cfPWV, 867 cm/s; age, 31 years) and relatively stiff (baPWV, 1700 cm/s; cfPWV, 1119 cm/s; age, 67 years) arteries, respectively.

Figure 3 shows examples of the relationship between cuff pressure and arterial volume during cuff deflation in one participant with a relatively compliant artery (baPWV, 1079 cm/s; cfPWV, 867 cm/s; age, 31 years), and in another with a relatively stiff artery (baPWV, 1700 cm/s; cfPWV, 1119 cm/s; age, 67 years). Arterial volume increased with cuff deflation, and the relationship between cuff pressure and arterial volume was described as a sigmoid curve for both individuals. The slope of the curve was steeper for the compliant than for the stiff artery. When the pressure-volume curves from both individuals were fitted to the equation F(x) = arctan (Bx + C) +D, the numerical coefficient B (API) was higher for the compliant than for the stiff artery (API, 0.0480 vs. 0.0276).

**Figure 4:**
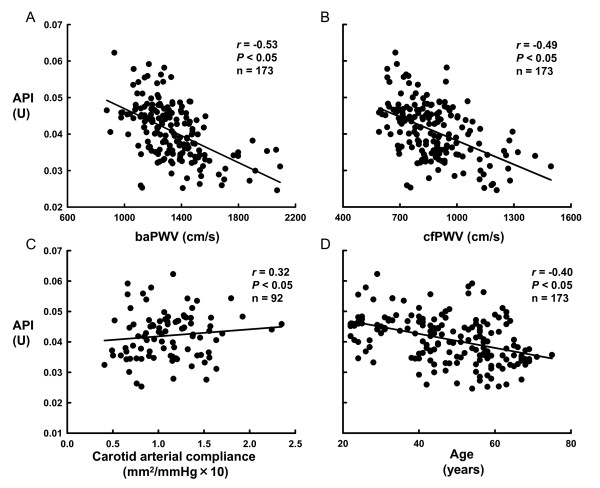
Relationships between new arterial stiffness index (Arterial Pressure-volume Index, API) and other arterial stiffness parameters or age. A. Relationship between brachial-ankle PWV (baPWV) and API. B. Relationship between carotid-femoral PWV (cfPWV) and API. C. Relationship between carotid arterial compliance and API. D. Relationship between age and API.

Figures 4A and 4B show the relationships between baPWV and API, and between cfPWV and API in all participants. We found that API was related to baPWV and cfPWV, and (baPWV, *r *= -0.53, *P *< 0.05; cfPWV, *r *= -0.49, *P *< 0.05). API was also related to carotid arterial compliance as shown in Figure 4C (*r *= 0.33, *P *< 0.05), and β-stiffness index (*r *= -0.34, *P *< 0.05). We also found that API was related to age as shown in Figure 4D (*r *= -0.40, *P *< 0.05).

A stepwise multiple regression analysis revealed that baPWV (β = -0.26) and carotid arterial compliance (β = 0.19) as well as MAP (β = -0.28) and the circumference of the upper arm (β = -0.21) were the independent determinants of API. On the contrary, API (β = -0.19) and MAP (β = 0.61) were the independent determinants of baPWV. API was also the independent determinants of carotid arterial compliance (β = 0.32).

## Discussion

We aimed to develop a method of evaluating arterial stiffness using oscillometric blood pressure measurement. The major new findings in this study were that a curve relationship between cuff pressure and arterial volume could be derived from blood pressure pulse obtained from oscillometric blood pressure measurement, and that after fitting an equation to the curve, a numerical coefficient of the equation (arterial stiffness index) was related to PWV(baPWV and cfPWV) and carotid arterial compliance. Furthermore, a stepwise multiple regression analysis demonstrated that baPWV and carotid arterial compliance were the independent determinants of API, and that API was the independent determinant of baPWV and carotid arterial compliance. These results suggest that arterial stiffness can be evaluated using our method based on oscillometric blood pressure measurement.

Only few methods have so far been proposed to assess arterial stiffness using oscillometric blood pressure measurement [23,24]. Liu et al. proposed a method for evaluating brachial arterial compliance using the oscillometric pulse [23]. Their method is based on oscillometric blood pressure measurement, but requires a record of not only the cuff pressure with a pressure transducer, but also the cuff volume with an air flow meter. The method examined by Sato et al. assesses arterial stiffness with evaluating the shape of the oscillometric envelope [24]. This method requires only cuff pressure recording, but the calculated index of arterial stiffness varies so that the measurement was repeated five times and the maximum and minimum values were excluded [24]. In the present study, we proposed a method that can assess arterial stiffness with recording only cuff pressure. Additionally, the coefficient of variation of replicate measurements at the experimental day for API was 7.5 ± 6%, which is comparable to that for cfPWV (7 - 8%) previously reported [19,25]. Furthermore, the day-to-day coefficient of variation for API in our pilot study was 6.0 ± 1.1%, whereas that for cfPWV and carotid arterial compliance in the previous studies were 5 - 9% [4,26]. Taken together, our method can assess arterial stiffness with recording only cuff pressure and the calculated arterial stiffness index (API) has reproducibility that is comparable to cfPWV and carotid arterial compliance.

It is possible that the slope of the pressure-volume curve in this study correlates with brachial artery stiffness. When blood pressure is measured oscillometrically, decreasing cuff pressure increases transmural pressure of the brachial artery wall, causing the brachial artery to distend and arterial volume to increase. Vessel distension corresponding to transmural pressure would be greater in a compliant than in a stiff brachial artery. Hence, the slope of the pressure-volume curve between cuff pressure and arterial volume would be steeper for those with a compliant brachial artery. This concept is in agreement with previous studies that have assessed relationships between changes in intra-arterial pressure and in corresponding vessel diameter using isolated arteries from humans and animals [11-17]. For example, the slope of the pressure-diameter curve of the atherosclerotic iliac artery is gentle and the calculated elastic modulus of the arterial wall increases in dogs fed with a high-cholesterol diet for 12 months [15]. Furthermore, a computer simulation that described the curve between transmural pressure and arterial volume during deflation of a cuff wrapped around the upper arm also found a steeper slope in compliant than in stiff brachial arteries [10]. According to the present and previous findings, the slope of the curve between the cuff pressure and arterial volume seems to vary depending on the stiffness (compliance) of the brachial artery. Thus, the arterial stiffness index (API) developed herein would reflect brachial arterial stiffness.

Although the API would reflect brachial arterial stiffness, it correlated with cfPWV, an arterial stiffness index of central arteries such as the aorta. One interpretation of this finding is that individuals with stiff (or compliant) central arteries also have stiff (or compliant) peripheral arteries. Arterial stiffness of the central arteries increases with advancing age [4,11,12,14], whereas the effect of age on the peripheral arteries is still controversial. However, API that is derived from brachial artery pulses was negatively correlated with age in the present study (Figure 4D). In agreement with this finding, the large peripheral arteries of the arms and legs as well as the aorta stiffen with age [27], although arterial stiffness with age is relatively modest in the peripheral arteries compared with the central arteries [27,28]. Different from the modest effects of age, regular endurance exercise in humans increases both femoral and carotid arterial compliance [29] and a high cholesterol diet in dogs increases iliac and carotid arterial stiffness [15]. It was noted that the effects of the exercise and high cholesterol diet on compliance/stiffness of femoral and iliac artery were greater than that of carotid artery [15,29]. Collectively, aging and lifestyle such as exercise and diet alters peripheral and central arterial stiffness in parallel, although the effects of age on peripheral artery might be modest, and thus API might be correlated with cfPWV.

API was correlated with PWV and carotid arterial compliance but the correlation coefficients were relatively low. We consider that the low correlation coefficients are probably due to the methodology differences rather than the poor validity of API. Indeed, the correlation coefficients between carotid arterial compliance and baPWV or cfPWV were also relatively low in the present study (*r *= -0.22; *r *= -0.14), despite the accepted methods clinically and experimentally. One explanation for the law correlations among methods is that each method does not entirely assess the same artery. Additionally, each approach may partly evaluate the same property of the arterial wall, but may also assess the different arterial properties. These might affect the correlation coefficients among API and PWV or carotid arterial compliance.

Our developed method has important clinical implications. We developed an arterial stiffness index based on oscillometric blood pressure measurements. If the algorithm to calculate the index was added to an oscillometric device, blood pressure and arterial stiffness could be simply and simultaneously measured. This would enable the early detection of arterial stiffness and thus contribute to the prevention of cardiovascular disease. Additionally, the API correlated with cfPWV, which is a stiffness index of the central artery. To assess central arterial stiffness is clinically important because it is a predictor of cardiovascular mortality [30,31]. The correlation between cfPWV and API suggests that the developed index could be used as a tool to screen central arterial stiffness. Furthermore, our developed methodology would be useful for daily control of arterial stiffness. Endurance exercise training for 2-3 months improved arterial stiffness [4-6], suggesting that arterial stiffness could be controlled in daily life. The developed method would match this demand because it is simple and easy for use.

The potential limitations in this study should be discussed. Firstly, we evaluated arterial volume indirectly via the oscillometric cuff pressure. Because, the muscles and the fat exist between the brachial blood vessel and the cuff, the muscle or fat size may affect on our estimated arterial volume, thereby API. Indeed, a stepwise multiple regression analysis revealed that the circumference of the upper arm was an independent determinant of API. This should be taken into account to use API. Secondly, we did not evaluate arterial stiffness among individuals with diseases that are related to this condition such as diabetes. Further studies are needed to examine whether our method can detect arterial stiffness in such patients.

## Conclusions

We developed a method of evaluating arterial stiffness by assessing the curve between cuff pressure and arterial volume using oscillometric blood pressure measurement. The pressure-volume curve was fitted using an equation and we identified a numerical coefficient of the equation as an index of arterial stiffness. The developed index was related to PWV (baPWV and cfPWV) and carotid arterial compliance.

## List of abbreviations

API: Arterial Pressure-volume Index; baPWV: Brachial-ankle PWV; cfPWV: Carotid-femoral PWV; Tcf: The time intervals between the carotid and femoral arterial pressure wave; Tba: The time between the brachial and post-tibial arterial pressure wave; Dcf: The path length from the carotid to the femoral artery; Dhb: The path lengths from the heart to the brachial artery; Dhf: The path lengths from the heart to the femur; Dfa: The path lengths from the femur to the ankle; MAP: Mean arterial blood pressure.

## Competing interests

The authors declare that they have no competing interests.

## Authors' contributions

HK conceived the study, performed data acquisition and analysis, and drafted the manuscript. YA contributed to the concept of the study, evaluated the data, and performed data analysis. TY contributed to the design of the study and interpretation of the data. MY recruited subjects and performed data acquisition and analysis. All authors read and approved the final manuscript.
